# Cancer-Specific Mortality in Rare Histological Subtypes of Prostate Cancer: Radical Prostatectomy Versus Radiation Therapy

**DOI:** 10.1245/s10434-026-19245-5

**Published:** 2026-02-20

**Authors:** Carolin Siech, Mario de Angelis, Letizia Maria Ippolita Jannello, Francesco Di Bello, Natali Rodriguez Peñaranda, Jordan A. Goyal, Fred Saad, Shahrokh F. Shariat, Salvatore Micali, Nicola Longo, Ottavio de Cobelli, Alberto Briganti, Mike Wenzel, Philipp Mandel, Luis A. Kluth, Felix K. H. Chun, Pierre I. Karakiewicz

**Affiliations:** 1 Cancer Prognostics and Health Outcomes Unit, Division of Urology, University of Montréal Health Center, Montréal, QC Canada; 2 Department of Urology, Goethe University Frankfurt, University Hospital, Frankfurt am Main, Germany; 3 Division of Experimental Oncology/Unit of Urology, URI, IRCCS Ospedale San Raffaele, Milan, Italy; 4 Vita-Salute San Raffaele University, Milan, Italy; 5 Department of Urology, IEO European Institute of Oncology, IRCCS, Milan, Italy; 6 Università degli Studi di Milano, Milan, Italy; 7 Department of Neuroscience, Science of Reproduction and Odontostomatology, University of Naples Federico II, Naples, Italy; 8 Department of Urology, AOU di Modena, University of Modena and Reggio Emilia, Modena, Italy; 9 Department of Urology, Comprehensive Cancer Center, Medical University of Vienna, Vienna, Austria; 10 Department of Urology, Weill Cornell Medical College, New York, NY USA; 11 Department of Urology, University of Texas Southwestern Medical Center, Dallas, TX USA; 12 Hourani Center for Applied Scientific Research, Al-Ahliyya Amman University, Amman, Jordan; 13 Department of Oncology and Haemato-Oncology, Università degli Studi di Milano, Milan, Italy

**Keywords:** Cancer-specific survival, External-beam radiotherapy, Rare diseases, SEER, Variant histology

## Abstract

**Background:**

Cancer-specific mortality (CSM) rates in patients with rare histological prostate cancer subtypes after treatment with radical prostatectomy (RP) versus radiation therapy (RT) are largely unknown.

**Methods:**

Relying on the Surveillance, Epidemiology, and End Results database (2004–2020), we identified patients with five prostate cancer subtypes treated with RP or RT. Kaplan–Meier analyses and Cox regression models addressed CSM.

**Results:**

Of 427,055 patients, 425,692 (99.68%) harbored acinar, 855 (0.20%) ductal, 324 (0.08%) mucinous, 54 (0.01%) signet ring cell adenocarcinoma, and 130 (0.03%) neuroendocrine carcinoma. Of those, 250,910 (59%), 592 (69%), 262 (81%), 34 (63%), and 34 (26%) were treated with RP, respectively. Five-year cancer-specific survival rates after RP versus RT were 99.2 versus 97.1% in acinar; 96.3 versus 87.1% in ductal; 98.7 versus 92.1% in mucinous; 97.0 versus 94.7% in signet ring cell; and 59.4 versus 20.5% in neuroendocrine carcinoma. In univariable Cox regression models, RP was associated with a lower CSM rate in acinar (hazard ratio [HR] 0.28; *p *< 0.001), ductal (HR 0.25; *p *< 0.001), and neuroendocrine (HR 0.36; *p *< 0.001), but not in mucinous (*p *= 0.052) and signet ring cell carcinoma (*p *= 0.8). After multivariable adjustment, RP remained an independent predictor of lower CSM in acinar (HR 0.35; *p *< 0.001), ductal (HR 0.30; *p* < 0.001), and neuroendocrine carcinoma (HR 0.53; *p* = 0.042).

**Conclusions:**

Higher CSM was recorded after RT in acinar, ductal, and neuroendocrine carcinoma. Conversely, no differences in CSM were identified when RP was compared with RT in mucinous and signet ring cell adenocarcinoma.

Acinar adenocarcinoma represents the dominant histology in patients with newly diagnosed prostate cancer (PCa).^1–5^ However, some patients may harbor rare histological subtypes of PCa, including ductal, mucinous, or signet ring cell adenocarcinoma as well as neuroendocrine carcinoma.^1–6^ Rare histological subtypes of PCa have been associated with differences in tumor biology, treatment response, and clinical outcomes, raising concern that treatment paradigms derived from acinar adenocarcinoma may not be universally applicable.^1,7–11^ Radical prostatectomy (RP) and radiation therapy (RT) represent well-established curative treatment options in patients with non-metastatic acinar adenocarcinoma of the prostate.^12,13^ In all these rare histological subtypes, the distinction between RP and RT as initial treatment modality has not received broad attention.^9–11^ Uncertainty about cancer control outcomes may apply when these competing treatment modalities are considered. In consequence, histology-specific analyses can be decisive for both clinicians in patient counseling and patients in clinical decision making between RP versus RT.

Based on this knowledge gap, we addressed cancer-specific mortality (CSM) rates according to treatment type, RP versus RT, across rare histological subtypes of PCa, namely ductal, mucinous, signet ring cell, and neuroendocrine carcinoma. In addition, we compared these two treatment alternatives in the dominant histology, namely acinar adenocarcinoma. Based on the concept of null hypothesis, we hypothesized that no differences in CSM exist between RP- versus RT-treated patients, reflecting the lack of definitive prior evidence favoring either modality. To test this hypothesis in a population-based cohort of patients with PCa treated in the United States, we relied on the Surveillance, Epidemiology, and End Results database (SEER 2004–2020).

## Materials and Methods

### Data Source and Study Population

**Fig. 1:**
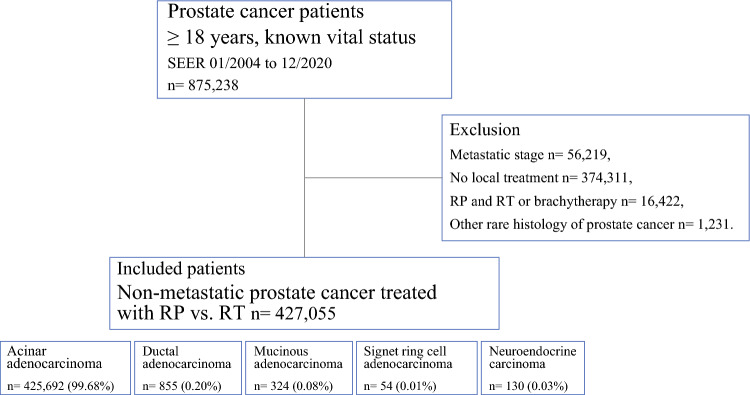
Consort diagram. RP, radical prostatectomy; RT, radiation therapy; SEER, Surveillance, Epidemiology, and End Results database

Within the SEER database (https://seer.cancer.gov/data/), which provides cancer statistics covering approximately 47.9% of the US population,^14^ we identified patients with histologically confirmed PCa (International Classification of Diseases [ICD], Tenth Revision site code C61). Specifically, we included histology types ranging from acinar (ICD for Oncology [ICD-O-3] codes 8140/3, 8550/3, and 8551/3) to ductal (ICD-O-3 codes 8201/3, 8260/3, 8380/3, 8500/3, 8501/3, 8503/3, 8521/3, 8523/3, and 8552/3), mucinous (ICD-O-3 codes 8480/3, and 8481/3), signet ring cell adenocarcinoma (ICD-O-3 code 8490/3), and finally to neuroendocrine carcinoma (ICD-O-3 codes 8013/3, 8020/3, 8041/3, 8045/3, 8240/3, 8244/3, and 8246/3). Within neuroendocrine, small-cell carcinoma was included, unlike in some previous reports where small-cell histology was analyzed in isolation.^10,11^ All other histological subtypes were excluded. Only patients aged ≥ 18 years, with non-metastatic stage (M0), known vital status, and known cause of death were included. Autopsy- or death-certificate-only cases were excluded. All included patients were treated with either RP (surgery site code 50) or external-beam RT. Patients receiving the combination of RP and RT or the combination of RT and brachytherapy were not considered (Figure 1). All missing data were marked as “unknown” in the present analyses.

All analyses and their reporting followed the SEER reporting guidelines. Due to the anonymously coded design of the SEER database, study-specific institutional review board ethics approval was not required. The study was conducted in accordance with the principles set in the Helsinki Declaration.

### Statistical Analyses

The primary study endpoints consisted of CSM, defined as all deaths due to PCa in accordance with the SEER mortality code. Three analytical steps were completed. First, baseline characteristics were tabulated to ascertain differences in baseline patient characteristics according to treatment with RP versus RT. Descriptive statistics included medians and interquartile ranges [IQR] for continuously coded variables, and Wilcoxon’s rank sum test examined the statistical significance of medians’ differences. Frequencies and proportions were recorded for categorical variables and Pearson’s chi-squared test assessed the statistical significance in proportions’ differences.

Second, Kaplan–Meier survival plots displayed CSM-free survival of patients with PCa treated with RP versus RT. Subsequently, univariable and multivariable Cox regression models were fitted to test for CSM differences according to RP versus RT. Adjustment variables consisted of prostate-specific antigen (PSA: < 10 vs. 10–20 vs. ≥ 20 ng/ml vs. unknown), clinical tumor stage (cTstage: cT1 vs. cT2 vs. cT3/4 vs. cTx), and Gleason sum score at biopsy (6 vs. 7 vs. ≥ 8 vs. unknown) when applicable. All analyses were separately performed in each PCa histological subtype (acinar vs. ductal vs. mucinous vs. signet ring cell adenocarcinoma vs. neuroendocrine carcinoma). Because of high rates of chemotherapy use even in localized stage neuroendocrine carcinoma, separate subgroup analyses addressed patients with neuroendocrine carcinoma histology after stratification according to chemotherapy use.

Analyses and reporting followed the STROBE guidelines for reporting observational studies.^15^ R software environment was used for statistical computing and graphics (R version 4.3.2; R Foundation for Statistical Computing, Vienna, Austria).^16^ Statistical tests were two sided with a level of significance set at *p *< 0.05.

## Results

### Descriptive Characteristics

Of 427,055 patients, 425,692 (99.68%) harbored acinar adenocarcinoma, 855 (0.20%) harbored ductal adenocarcinoma, 324 (0.08%) harbored mucinous adenocarcinoma, 54 (0.01%) harbored signet ring cell adenocarcinoma, and 130 (0.03%) harbored neuroendocrine carcinoma (Figure 1). The rates of RP ranged from 26% in neuroendocrine to 59% in acinar, 63% in signet ring cell, 69% in ductal, and 81% in mucinous carcinoma. Among RP-treated patients, rates of pelvic lymph node dissection ranged from 65% in acinar to 74% in mucinous, 80% in ductal, 85% in neuroendocrine, and 88% in signet ring cell carcinoma. Of those treated with both RP and pelvic lymph node dissection, the median number of removed lymph nodes ranged from five (IQR 3–10) in acinar to seven (IQR 4–13) in mucinous, seven (IQR 4–15) in neuroendocrine, eight (IQR 4–14) in ductal, and eight (IQR 4–18) in signet ring cell carcinoma.

In general, RP versus RT patients were younger, more frequently exhibited PSA < 10 ng/ml, and less frequently exhibited cT3/cT4 stage (Table 1). Conversely, no clinically meaningful differences were observed for race/ethnicity, Gleason sum score at biopsy, and cNstage.

**Table 1:** Descriptive characteristics of patients with acinar adenocarcinoma and rare histological subtypes of prostate cancer, stratified according to treatment type: radical prostatectomy (RP) vs. radiation therapy (RT)

Characteristic	Acinar adenocarcinoma		Ductal adenocarcinoma		Mucinous adenocarcinoma		Signet ring cell adenocarcinoma		Neuroendocrine carcinoma	
	RP (n = 250,910; 59%)	RT (n = 174,782; 41%)	RP (n = 592; 69%)	RT (n = 263; 31%)	RP (n = 262; 81%)	RT (n = 62; 19%)	RP (n = 34; 63%)	RT (n = 20; 37%)	RP (n = 34; 26%)	RT (n = 96; 74%)
Age, years	62 (57–67)	69 (64–75)	65 (59–69)	74 (69–79)	60 (54–66)	67 (63–77)	65 (60–68)	70 (66–74)	66 (62–70)	72 (64–79)
Race/ethnicity										
Caucasian	181,136 (72)	117,401 (67)	408 (69)	193 (73)	187 (71)	47 (76)	21 (62)	14 (70)	27 (79)	76 (79)
Hispanic	24,812 (10)	17,002 (10)	56 (10)	23 (9)	41 (15)	7 (11)	4 (12)	4 (20)	4 (12)	8 (8)
African American	30,357 (12)	28,321 (16)	69 (11)	28 (11)	17 (7)	6 (10)	6 (18)	2 (10)	2 (6)	9 (8)
Other	14,605 (6)	12,058 (7)	59 (10)	19 (7)	17 (7)	2 (3)	3 (8)	0 (0)	1 (3)	3 (3)
PSA, ng/mL										
<10	184,714 (74)	108,389 (62)	409 (70)	155 (59)	173 (66)	35 (56)	17 (50)	9 (45)	19 (56)	47 (49)
10–20	31,113 (12)	36,033 (21)	99 (17)	48 (18)	48 (18)	7 (11)	7 (21)	3 (15)	10 (29)	14 (15)
≥20	9805 (4)	19,302 (11)	31 (5)	29 (11)	17 (7)	8 (13)	9 (26)	4 (20)	2 (6)	9 (9)
Unknown	25,278 (10)	11,058 (6)	53 (9)	31 (12)	24 (9)	12 (19)	1 (3)	4 (20)	3 (9)	26 (27)
Gleason sum score biopsy										
6	47,882 (19)	26,538 (15)	49 (8)	8 (3)	18 (7)	4 (6)	2 (6)	0 (0)	–	–
7	78,436 (31)	56,624 (32)	174 (29)	30 (11)	92 (35)	13 (21)	3 (9)	0 (0)	–	–
≥8	25,028 (10)	32,522 (19)	211 (37)	127 (49)	36 (14)	16 (26)	13 (38)	8 (40)	–	–
Unknown	99,564 (40)	59,098 (34)	158 (27)	98 (37)	116 (44)	29 (47)	16 (47)	12 (60)	–	–
cTstage										
cT1	134,255 (54)	107,980 (62)	217 (37)	107 (41)	130 (50)	32 (52)	14 (42)	10 (50)	7 (21)	10 (10)
cT2	46,831 (19)	33,262 (19)	136 (23)	60 (23)	57 (22)	11 (18)	9 (26)	5 (25)	13 (38)	10 (10)
cT3/cT4	8102 (3)	8402 (5)	47 (8)	57 (22)	12 (4)	7 (11)	2 (6)	2 (10)	7 (21)	57 (59)
cTx	61,722 (25)	25,138 (14)	192 (32)	39 (15)	63 (24)	12 (19)	9 (26)	3 (15)	7 (21)	19 (20)
cNstage										
cN0	222,816 (89)	164,978 (94)	482 (81)	244 (93)	230 (88)	53 (85)	27 (79)	16 (80)	22 (65)	53 (55)
cN1	5872 (2)	2841 (2)	29 (5)	10 (4)	5 (2)	2 (3)	4 (12)	3 (15)	7 (21)	30 (31)
cNx	22,222 (9)	6963 (4)	81 (14)	9 (3)	27 (10)	7 (11)	3 (9)	1 (5)	5 (15)	13 (14)

Data are presented as median (interquartile range) or n (%) unless otherwise indicated

cNstage = clinical lymph node stage; cTstage = clinical tumor stage; PSA = prostate-specific antigen; RP = radical prostatectomy; RT = radiation therapy

### Kaplan–Meier Survival Analyses Addressing Cancer-Specific Survival

**Fig. 2:**
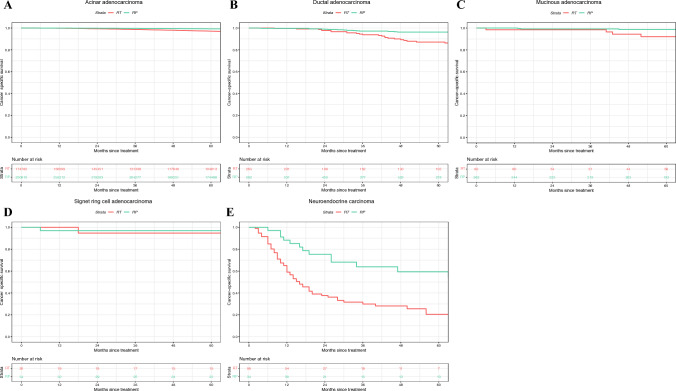
Kaplan–Meier survival analyses addressing cancer-specific survival in patients with **A** acinar, **B** ductal, **C** mucinous, **D** signet ring cell, and **E** neuroendocrine carcinoma, stratified according to treatment type: radical prostatectomy (RP) versus radiation therapy (RT)

After RP versus RT, 5-year CSM-free survival rates were 99.2 versus 97.1% in acinar (Δ 2.1%; *p *< 0.001), 96.3 versus 87.1% in ductal (Δ 9.2%; *p* < 0 .001), 98.7 versus 92.1% in mucinous (Δ 6.6%; *p* = 0.037), 97.0 versus 94.7% in signet ring cell (Δ 2.3%; *p *= 0.8), and 59.4 versus 20.5% in neuroendocrine PCa (Δ 38.9%; *p *< 0.001; Figure 2).

### Cox Regression Models Addressing Cancer-Specific Mortality

The above rates resulted in a univariable CSM hazard ratio (HR) of 0.28 for acinar adenocarcinoma (95% confidence interval [CI] 0.27–0.30; *p *< 0.001); 0.25 for ductal adenocarcinoma (95% CI 0.15–0.40; *p* < 0.001); 0.27 for mucinous adenocarcinoma (95% C 0.07–1.01; *p *= 0.052); 1.31 for signet ring cell adenocarcinoma (95% CI 0.24–7.15; *p *= 0.8); and 0.36 for neuroendocrine carcinoma (95% CI 0.20–0.65; *p *< 0.001;Table 2). After multivariable adjustment for PSA, cTstage, and Gleason sum score at biopsy when applicable, the multivariable CSM HR increased to 0.35 for acinar adenocarcinoma (95% CI 0.34–0.36; *p* < 0.001), 0.30 for ductal adenocarcinoma (95% CI 0.18–0.50; *p *< 0.001), and 0.53 for neuroendocrine carcinoma (95% CI 0.28–0.98; *p* = 0.042). The low number of observations and events meant that multivariable testing could not be applied to mucinous and signet ring cell histological subtypes.

**Table 2:** Univariable Cox regression models addressing cancer-specific mortality in patients with acinar adenocarcinoma and rare histological subtypes of prostate cancer, according to treatment type: radical prostatectomy (RP) vs. radiation therapy (RT)

RP vs. RT	HR (95% CI)	p-Value
Acinar adenocarcinoma	0.28 (0.27–0.30)	**<0.001**
Ductal adenocarcinoma	0.25 (0.15–0.40)	**<0.001**
Mucinous adenocarcinoma	0.27 (0.07–1.01)	0.052
Signet ring cell adenocarcinoma	1.31 (0.24–7.15)	0.8
Neuroendocrine carcinoma	0.36 (0.20–0.65)	**<0.001**

Bold values indicate statistically significant p-values based on a level of significance of 0.05

CI, confidence interval; HR, hazard ratio; RP, radical prostatectomy; RT, radiation therapy

### Subgroup Analyses of Patients with Neuroendocrine Carcinoma after Stratification According to Chemotherapy Use

Of 130 patients with neuroendocrine carcinoma histology, 82 (63%) received chemotherapy. Of these, 68 (83%) were treated with RT and 14 (17%) with RP. Conversely, of 48 patients with neuroendocrine carcinoma histology who did not receive chemotherapy, 28 (58%) were treated with RT and 20 (42%) with RP. After RP versus RT, 5-year CSM-free survival rates were 30.6 versus 27.4% in patients who received chemotherapy (*p* = 0.6) and 79.1% versus not reached in patients who did not receive chemotherapy (*p *< 0.001). The above rates resulted in a univariable CSM HR of 0.81 for chemotherapy-treated patients (95% CI 0.40–1.67; *p* = 0.6) and 0.11 (95% CI 0.04–0.33; *p* < 0.001) for patients who did not receive chemotherapy. The limited number of observations and events meant that multivariable adjustment was not feasible in subgroup analyses.

## Discussion

Survival outcomes for PCa after treatment with RP or RT are largely based on patients with acinar adenocarcinoma.^12,13,17–21^ In consequence, uncertainty about cancer control outcomes may apply when these competing treatment modalities are considered in rare histological subtypes of PCa.^9,10^ We addressed this knowledge gap and hypothesized that no differences in CSM exist between RP- versus RT-treated patients. Relying on a contemporary population-based cohort of patients with PCa in the USA (SEER 2004–2020), we made several noteworthy observations.

First, PCa histological subtypes represent rare primaries.^1,2,6^ Specifically, Marcus et al.^1^ relied on a study period of 36 years (SEER 1973–2008) and identified 662 patients with ductal, 806 with mucinous, 130 with signet ring cell adenocarcinoma, and 502 with neuroendocrine carcinoma among 793,064 patients with PCa across all tumor stages. These sample sizes accounted for, respectively, 0.08, 0.10, 0.02, and 0.06% of all patients with PCa newly diagnosed between 1973 and 2008. In the current study, we focused on the most contemporary cohort of patients with non-metastatic PCa treated with RP or RT (SEER 2004–2020) and identified 855 (0.20%) ductal, 324 (0.08%) mucinous, 54 (0.01%) signet ring cell adenocarcinoma, and 130 (0.03%) neuroendocrine carcinomas. In summary, PCa with histological subtypes accounted for <1% in our study cohort. Single- or multi-institutional series addressing PCa with histological subtypes relied on substantially smaller sample sizes.^22–28^ In consequence, patients with PCa with histological subtypes should ideally be examined in large population-based analyses, as was done in the current study, when cancer-control outcomes represent the outcome of interest.

Second, we identified important differences in baseline characteristics between patients treated with RP versus RT across all five examined PCa subtypes. In general, RP patients more frequently exhibited PSA < 10 ng/ml and less frequently exhibited cT3/cT4 stage than their counterparts treated with RT. Similar differences in baseline characteristics have previously been recorded for patients with acinar and ductal adenocarcinoma.^9,13,19,29^ To the best of our knowledge, we are the first to compare the characteristics of patients with mucinous and signet ring cell adenocarcinoma as well as neuroendocrine carcinoma treated with either RP or RT. Therefore, no direct comparison is possible for these PCa subtypes. However, it is plausible that disease aggressiveness might affect treatment choice. Accordingly, it is essential to adjust for these covariates in multivariable models, as was done in the current study.

Third, we addressed CSM as cancer-control endpoints. In survival analyses, we tested for differences in five PCa subtypes treated with either RP versus RT. Specifically, differences in 5-year CSM-free survival rates according to treatment with RP versus RT ranged from 38.9% in neuroendocrine carcinoma (*p *< 0.001) to 9.2% in ductal adenocarcinoma (*p *< 0.001), 6.6% in mucinous adenocarcinoma (*p* = 0.037), 2.3% in signet ring cell adenocarcinoma (*p *= 0.8), and to 2.1% in acinar adenocarcinoma (*p *< 0.001). In Cox regression models, these rates translated into HRs of 0.36 for neuroendocrine carcinoma (*p *< 0.001), 0.25 for ductal (*p* < 0.001), 0.27 for mucinous (*p* = 0.052), 1.31 for signet ring cell (*p* = 0.8), and 0.28 for acinar adenocarcinoma (*p* < 0.001). The limited numbers of observations and events in mucinous and signet ring cell adenocarcinoma meant that multivariable adjustment for tumor characteristics could only be performed in Cox regression models addressing acinar and ductal adenocarcinoma as well as neuroendocrine carcinoma. In these multivariable models, RP was invariably associated with more favorable CSM rates in ductal (HR 0.30; *p *< 0.001), acinar (HR 0.35; *p* < 0.001), and neuroendocrine carcinoma (HR 0.53; *p* = 0.042). To the best of our knowledge, previous population-based analyses only examined cancer-control outcomes after RP versus RT in acinar, ductal, or small-cell carcinoma of the prostate.^9–11,17,19^ Unfortunately, analyses addressing small-cell carcinoma of the prostate within the National Cancer Database (NCDB) could only address overall survival as a study endpoint.^10,11^ Therefore, direct comparison of survival rates is not possible. However, in localized-stage neuroendocrine carcinoma, cancer-specific deaths accounted for 74% of all deaths recorded at 5 years of follow-up.^7^ In addition, no previous study examined CSM rates in patients with mucinous or signet ring cell adenocarcinoma after treatment with either RP or RT. In consequence, these findings cannot be directly compared with any previous study. Differences in CSM rates after treatment with RP versus RT in patients with ductal adenocarcinoma and neuroendocrine carcinoma may be explained in several ways. First, CSM differences may reflect variations in tumor biology, such as immunohistochemical profile and more advanced stage at initial diagnosis.^30,31^ Second, differences in outcomes may be related to variances in treatment sensitivity, such as relative resistance to radiation and androgen-directed therapies, suggesting a potential advantage for RP in achieving cancer control.^32^ These biological differences may be accompanied by variability in patient selection (e.g. based on patient age). These explanations remain speculative but do highlight the need for histology-specific treatment considerations and to further evaluate the underlying biology.

Taken together, our results demonstrate the rarity of ductal, mucinous, signet ring cell adenocarcinoma as well as neuroendocrine carcinoma of the prostate. These histological subtypes collectively account for <1% of the study cohort. In survival analyses, treatment with RP was invariably associated with more favorable CSM in patients with acinar, ductal, or neuroendocrine carcinoma compared with RT. Conversely, CSM rates after RP versus RT did not show statistically significant or clinically meaningful differences in mucinous or signet ring cell adenocarcinoma. The above observations may help clinicians in decision-making between RP and RT. Nonetheless, they should ideally be validated within equally large or even larger population-based databases. Additionally, causal mechanisms between treatment type and CSM require further elucidation in subsequent studies.

Despite its novelty, the present study is not devoid of limitations. First, the current study uses a retrospective and observational study design. Even after systematic adjustment for biases and confounders in multivariable models, a potential for selection biases remained. Specifically, selection biases may be based on patient (e.g. age) or tumor characteristics (e.g. cTstage and cNstage). This limitation is shared with all previous analyses relying on retrospective databases, such as the SEER database^1–3,7,9,12,13,17–19,33,34^ or the NCDB.^5,10,11,20,21,29^ Nevertheless, the association between treatment type and survival outcomes reported within the present study should be interpreted cautiously. Prospective randomized studies are required to validate the observed findings and to explore causal pathways. However, given the rarity of histological subtypes of PCa, it appears unlikely that a prospective randomized trial addressing the treatment comparison (RP vs. RT) will be completed. Second, despite the large scale of the SEER database, the number of patients with PCa histological subtypes within the SEER database is limited because of the rarity of ductal, mucinous, signet ring cell adenocarcinoma, as well as neuroendocrine carcinoma of the prostate. The low number of observations and events meant that multivariable analyses could not be completed for mucinous and signet ring cell histological subtypes, limiting the generalizability and interpretability of our results. In addition, subgroup analyses, such as comparisons of small- versus non-small-cell neuroendocrine carcinomas were not possible. Moreover, patients with even rarer histological subtypes of PCa, such as sarcomatoid and adenosquamous carcinoma, could not be included in the current study because numbers were insufficient. Third, ICD-O-3 histology codes originate from patient records and were not validated by central review. Furthermore, we could not distinguish between pure versus mixed rare histological subtypes of PCa. In general, histological subtypes are coded only if they account for at least 50% of the tumor, which can underreport rare histological subtypes. This limitation is particularly relevant in biopsy-based diagnoses, where limited tissue sampling may fail to capture focal or heterogeneous histological features. In contrast, RP specimens provide more tissue than diagnostic biopsies, increasing the likelihood of identifying rare histological subtypes. Consequently, differences in tissue sampling between treatment groups may affect histological subtype detection.

Fourth, the current study focuses on the comparison of RP versus RT as they represent the most common active treatment options for non-metastatic PCa. Other treatment strategies, such as active surveillance or brachytherapy, which may also be considered in select patients with non-metastatic PCa were not included.^19^ Furthermore, the current version of the SEER (2004–2020) database does not provide any information regarding the use of androgen deprivation therapy and the frequency and dosage of radiotherapy. In addition, detailed information addressing chemotherapy, such as type, duration or dosage, is unavailable. This limitation is particularly relevant for patients with neuroendocrine carcinoma, for whom chemotherapy is frequently administered, even in localized disease. Consequently, these treatment-related factors could not be accounted for in the present analyses. Residual treatment selection bias cannot be excluded. Finally, the SEER database does not provide earlier cancer-control endpoints than CSM. Therefore, other study endpoints that could be equally as interesting as CSM, such as biochemical recurrence or metastasis-free survival, could not be addressed within the current database.

## Conclusions

Higher CSM was recorded after RT in acinar, ductal, and neuroendocrine carcinoma. Conversely, no differences in CSM were identified when RP was compared with RT in mucinous and signet ring cell adenocarcinoma.
